# Fast data-driven learning of parallel MRI sampling patterns for large scale problems

**DOI:** 10.1038/s41598-021-97995-w

**Published:** 2021-09-29

**Authors:** Marcelo V. W. Zibetti, Gabor T. Herman, Ravinder R. Regatte

**Affiliations:** 1 Center for Biomedical Imaging, Department of Radiology, New York University School of Medicine, New York, NY 10016 USA; 2 Department of Computer Science, The Graduate Center, City University of New York, New York, NY 10016 USA

**Keywords:** Biomedical engineering, Computational science

## Abstract

In this study, a fast data-driven optimization approach, named bias-accelerated subset selection (BASS), is proposed for learning efficacious sampling patterns (SPs) with the purpose of reducing scan time in large-dimensional parallel MRI. BASS is applicable when Cartesian fully-sampled k-space measurements of specific anatomy are available for training and the reconstruction method for undersampled measurements is specified; such information is used to define the efficacy of any SP for recovering the values at the non-sampled k-space points. BASS produces a sequence of SPs with the aim of finding one of a specified size with (near) optimal efficacy. BASS was tested with five reconstruction methods for parallel MRI based on low-rankness and sparsity that allow a free choice of the SP. Three datasets were used for testing, two of high-resolution brain images ($\text {T}_{2}$-weighted images and, respectively, $\text {T}_{1\rho }$-weighted images) and another of knee images for quantitative mapping of the cartilage. The proposed approach has low computational cost and fast convergence; in the tested cases it obtained SPs up to 50 times faster than the currently best greedy approach. Reconstruction quality increased by up to 45% over that provided by variable density and Poisson disk SPs, for the same scan time. Optionally, the scan time can be nearly halved without loss of reconstruction quality. Quantitative MRI and prospective accelerated MRI results show improvements. Compared with greedy approaches, BASS rapidly learns effective SPs for various reconstruction methods, using larger SPs and larger datasets; enabling better selection of sampling-reconstruction pairs for specific MRI problems.

## Introduction

### Motivation

Magnetic resonance imaging (MRI) is one of the most versatile imaging modalities, it can provide answers to medical questions through the measurements of various properties of the resonant spins in the human body. Unfortunately, the more information we seek from MRI, the longer is the acquisition time^1,2^. This makes the acquisition of high-resolution three-dimensional (3D) volume imaging of the human body time-consuming. Shortening the scan time in MRI is necessary for capturing dynamic processes, quantitative measurements, and for reducing health-care costs and increasing patient comfort. One effective way to reduce scan time is through *undersampling*, in which only part of the total set of measurements, specified by a *sampling pattern* (SP), is acquired. This approach is also called *accelerated MRI.*

### The specific content of this paper

**Figure 1:**
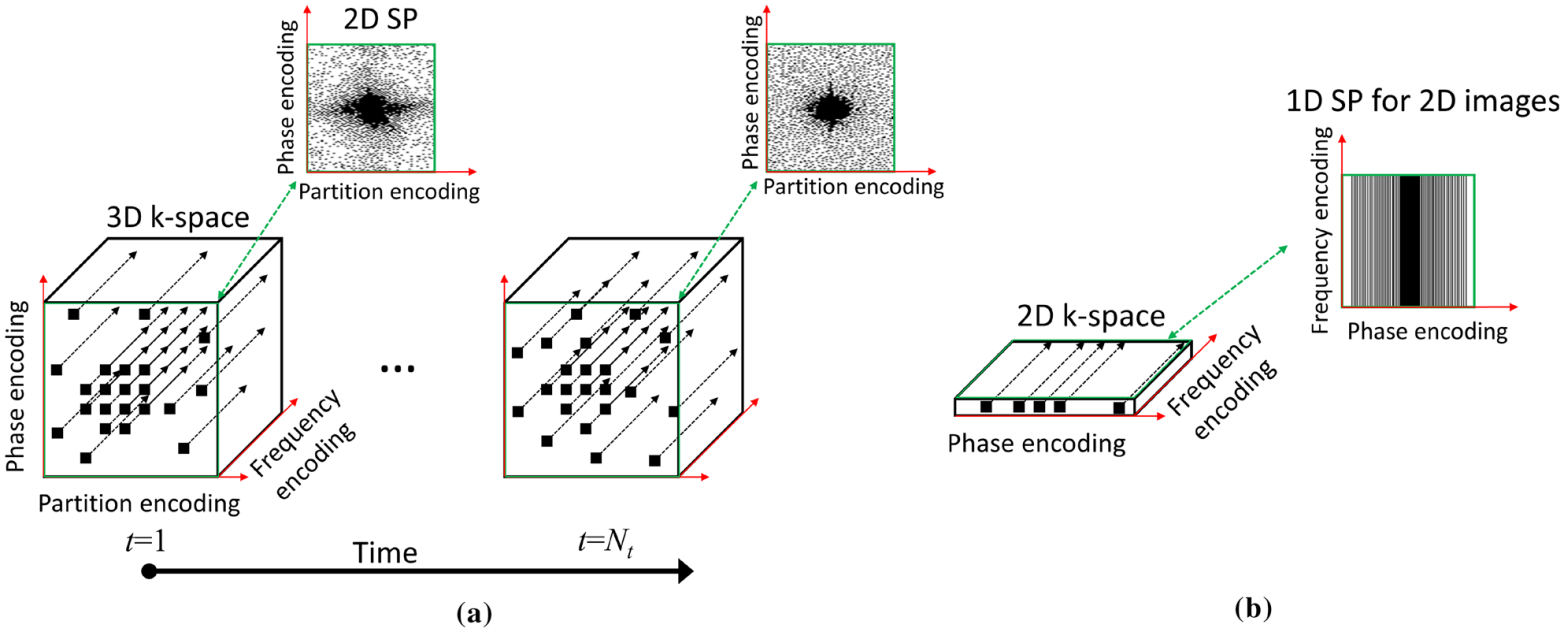
Illustration of the (**a**) 3D + time data collection scheme considered in this work. The sampling pattern is in 2D + time, it comprises the time-varying phase and partition encoding positions, for each of which data are to be collected by the MRI scanner for all frequency encoding positions. Our method can also be applied to (**b**) 2D data collection with fully-sampled lines in the frequency-encoding direction and a 1D sampling pattern denoting phase encoding positions.

We propose and validate a new *data-driven optimization* (DDO) approach to learn the SP in parallel MRI applications. Our focus is on Cartesian 3D high-resolution and 3D quantitative MRI problems in which the data are collected along multiple k-space lines (in the frequency-encoding direction) with the SP a 2D (phase/partition-encoding directions) entity, as illustrated in Fig. 1a. Reconstruction may be be performed as a fully 3D process, but we assume that a Fourier transform is applied in the frequency-encoding direction and the volume is separated into multiple slices for 2D reconstructions. We also tested the method with smaller-size 1D (phase-encoding) SP used in Cartesian 2D acquisitions, as illustrated in Fig. 1b.

The proposed approach is applicable to any parallel MRI method that allows a free selection of the SP, such as compressed sensing (CS)^3–6^ and low-rank approaches. Methods that directly recover the k-space elements, such as simultaneous auto-calibrating and k-space estimation (SAKE)^7^, low-rank modeling of local k-space neighborhoods (LORAKS)^8^, generic iterative re-weighted annihilation filter (GIRAF)^9^, and annihilating filter-based low-rank Hankel matrix approach (ALOHA)^10^, among others, can be used. We tested the proposed optimization approach for P-LORAKS^11^ and three different multi-coil CS approaches with different priors^12^. The contribution of the proposed approach is a new learning algorithm, named bias-accelerated subset selection (BASS), that can optimize large sampling patterns, using large datasets, spending significantly less processing times as compared to previous approaches. Moreover, the SPs optimized by BASS can achieve good image quality with short acquisition times, improving clinical tasks. A very preliminary presentation of our approach is in^13^.

### Background and purpose

Fast magnetic resonance (MR) pulse sequences for measurements acquisition^1,2,14^, parallel imaging (PI) using multichannel receive radio frequency arrays^15–17^, and CS^3–6^ are examples of advancements towards rapid MRI. PI uses multiple receivers with different spatial coil sensitivities to capture samples in parallel^18^, increasing the amount of measurements in the same scan time. Further, undersampling can be used to reduce the overall scan time^15–17^. CS relies on incoherent sampling and sparse reconstruction. With incoherence, the sparse signals spread almost uniformly in the sampling domain, and random-like patterns can be used to undersample the k-space^3–5,19,20^.

Successful reconstructions with undersampled measurements, such as PI and CS, use prior knowledge about the true signal to remove the artifacts of undersampling, preserving most of the desired signal. Essentially, the true signal is redundant and can be compactly represented in a certain domain, subspace, or manifold, of much smaller dimensionality^21,22^. Low-rank signal representation^23^ and sparse representation^24^, are two examples of this kind. Deep learning-based reconstructions have shown that undersampling artifacts can also be separated from true signals by learning the parameters of a neural network from sampled datasets^23,25,26^.

The quality of image reconstruction depends on the sampling process. CS is an example of how the SP can be modified^27–29^, compared to standard uniform sampling^30^, so as to be effective for a specific signal recovery strategy^29,31^. According to pioneering theoretical results^27,32,33^, restricted isometry properties (RIP) and incoherence are key for CS. In MRI, however, RIP and incoherence are more like guidelines for designing random sampling^3,5,29^ than target properties. New theoretical results^34,35^ revisited the effectiveness of CS in MRI; in particular, elucidating that incoherence is not a strict requirement. Also, studies^36,37^ show that SPs with optimally incoherent measurements^3^ do not achieve the best reconstruction quality, leaving room for effective empirical designs. SPs such as variable density^38–40^, Poisson disk^41,42^, or even a combination of both^43,44^ show good results in MRI reconstruction without relying on optimal incoherence properties.

In many CS-MRI methods, image quality improves when SP is learned utilizing a fully sampled k-space of similar images of particular anatomy as a reference^45–49^. Such adaptive sampling approaches adjust the probability of the k-space points of variable density SP according to the k-space energy of reference images^45–50^. Such SP design methods have been developed for CS reconstructions, but generally they do not consider the reconstruction method to be used.

Statistical methods for optimized design techniques can be used for finding best sampling patterns^51,52^. Experimental design methods, especially using optimization of Cramér-Rao bounds, are general and focus on obtaining improved signal-to-noise ratio (SNR). These approaches were used for fingerprinting^53^, PI^54^, and sparse reconstructions^51^. They do not consider specific capabilities of the reconstruction algorithm in the design of the SP, even though some general formulation is usually assumed.

In DDO approaches, the SP is optimized for reference images or datasets containing several images of particular anatomy, using a specific method for image reconstruction^55–59^. The main premise is that the optimized SP should perform well with other images of the same anatomy when the same reconstruction method is used. These approaches can be extended to jointly learning the reconstruction and the sampling pattern, as shown in^60–62^. DDO is applicable to any reconstruction method that accepts various SPs. In^56^, DDO for PI and CS-MRI is proposed, the selection of the SP is formulated as a subset selection problem^63,64^, which is solved using greedy optimization of an image domain criterion (an extension of^55^ for single-coil MRI); see also^57^ .

Finding an optimal SP via subset selection problem is, in general, an NP-hard problem. Also, each candidate SP needs to be evaluated on a large set of images, which may involve reconstructions with high computational cost. Effective minimization algorithms are fundamental for the applicability of these DDO approaches with large sampling patterns.

### Existent subset selection approaches for SP optimization

Commonly used in prior works are the greedy approaches; classified as forward^29,55,65^ (increase the number of points sampled in the SP, starting from the empty set), backward^51,65^ (reduce the number of points in the SP, from fully sampled), or hybrid^63^. Considering the current SP, greedy approaches test candidates SPs, that are one k-space element different, to be added (or removed). After testing, they add (or remove) the k-space element that provides the best improvement in the cost function^64^.

Greedy approaches have a disadvantage regarding computational cost because of the large number of evaluations or reconstructions. Assuming that fully-sampled k-space measurements are of size *N*, whereas the undersampled measurements are of size $M<N$, and there are $N_{i}$ images, or data items, used for the learning process, the greedy approach will take $N\times N_{i}$ reconstructions just to find the best first sampling element of the SP (not considering the next $M-1$ k-space elements that still have to be computed). This makes greedy approaches computationally unfeasible for large-scale MRI problems. As opposed to this, the approach proposed in this work can obtain a good SP using $50N_{i}$ to $500N_{i}$ image reconstructions (for all the *M* k-space elements of the SP).

The approach in^55^ is only feasible because it was applied to one-dimensional (1D) undersampling, such as in Fig. 1b, with a small number of images in the dataset and single-coil reconstructions. The approach was extended to 1D+time dynamic sequences^57^ and to parallel imaging^56^, but it requires too many evaluations, practically prohibitive for large datasets and large sampling patterns.

A different class of learning algorithms for subset selection^64^, not exploited yet by SP learning, use bit-wise mutation, such as Pareto optimization algorithm for subset selection (POSS)^64,66,67^. These learning approaches are less costly per iteration since they evaluate only one candidate SP and accept it if the cost function is improved. POSS is not designed for fast convergence, but for achieving good final results. However, these machine learning approaches can be accelerated if the changes are done smartly and effectively instead of randomly.

### Other fast approaches for DDO of SP

Besides the formulation of DDO of SP as a subset selection problem, other approaches have been investigated. The use of deep learning for image reconstruction^23,25,26,68^ have been extended to learning the SP. In^60^, a probabilistic sampling mask is learned inside the neural network, following by a random generation of SPs. In^62^, twice continuously differential models are used to find the SP for variational problems. While these approaches are also faster than^55^ to learn the SP, they are less flexible. The parallel MRI methods cited in the Section “The specific content of this paper” cannot be used, and only quadratic cost functions can be optimized. In^61,69,70^, parametric formulation of non-Cartesian k-space trajectories are optimized. While being interesting approaches, they cannot be applied to our Cartesian 3D problem described in “The specific content of this paper”. Another approach for improving image quality through better sampling is the use of active sampling^71–74^, in which the next k-space sampling positions are estimated during the acquisition using the data that have been captured. While promising, this approach requires significant changes within the MRI scanning sequence that are not widely available. As opposed to that, our current approach to find the best (optimized) fixed 3D Cartesian SP for a given anatomy, contrast, and pre-determined reconstruction method, can be included in an accelerated (compressed sensing and parallel) MRI scanning protocol, simply replacing an existent non-optimized SP. For this task, the subset selection formulation of DDO of the SP seems to be the most effective approach for our applications of interest.

## Theory

### Specification of our aim

Referring to Fig. 1, we use $\Gamma$ to denote the set of size *N* comprising in the Cartesian grid all possible (a) time-varying phase and partition encoding positions in the 3D + time data collection scheme or (b) all possible phase encoding positions in the 2D data collection scheme. Our instrument (a multi-coil MRI scanner) can provide measurements related to these sampling positions. Each such “measurement” comprises a fixed number (we denote it by $N_{s}$) of measurements values for k-space points, i.e. the points on a line in the frequency-encoding direction for all coils. The measurements for the *N* positions of $\Gamma$ are represented as the $N_{s}N$-dimensional complex-valued vector ${\mathbf {m}}$, these are referred to as *fully-sampled measurements*.

Let $\Omega$ be any subset (of size $M\le N$) of $\Gamma$; it is referred to as a *sampling pattern* (SP). The undersampled measurements of ${\mathbf {m}}$, restricted to *M* positions in $\Omega$, is represented as the $N_{s}M$-dimensional complex-valued vector

1 $$\begin{aligned} \bar{{\mathbf {m}}}={\mathbf {S}}_{\Omega }{\mathbf {m}}, \end{aligned}$$

where ${\mathbf {S}}_{\Omega }$ is a $N_{s}M\times N_{s}N$ matrix is referred to as the *sampling function*. Such $\bar{{\mathbf {m}}}$ is referred to as the undersampled measurements for the SP $\Omega$. The *acceleration factor* (AF) is defined as *N*/*M*. Note that, in practice, the reduction of the scan time depends on the pulse sequence used^2^. We assume here that the acquisition of $N_{s}$ measurements values for any element of $\Gamma$ requires the same scan time.

It is assumed that we have a defined *recovery algorithm* *R* that, for any SP $\Omega$ and any undersampled measurements $\bar{{\mathbf {m}}}$ for that SP, provides an estimate, denoted by $R(\Omega ,\bar{{\mathbf {m}}})$, of the fully-sampled measurements. A method for finding an efficacious choice $\Omega$ in a particular application area is our subject matter. Efficacy may be measured in the following data-driven manner.

Let $N_{i}$ be the number of images and also the number of fully sampled measurements items (denoted by ${\mathbf {m}}_{1},\ldots ,{\mathbf {m}}_{N_{i}}$, called the *training* *measurements*) used in the learning process to obtain an efficacious $\Omega$. Intuitively, we wish to find a SP $\Omega$ such that all the measurements ${\mathbf {m}}_{i}$, for $1\le i\le N_{i}$, are “near” to their respective recovered versions $R(\Omega ,{\mathbf {S}}_{\Omega }{\mathbf {m}}_{i})$ from the undersampled measurements. Using $f({\mathbf {m}},{\mathbf {n}})$ to denote the “distance” between two fully-sampled measurements ${\mathbf {m}}$ and ${\mathbf {n}}$, we define the *efficacy* of a SP $\Omega$ as:

2 $$\begin{aligned} F(\Omega )=\frac{1}{N_{i}}\sum _{i=1}^{N_{i}}f\left( {\mathbf {m}}_{i},R \left( \Omega ,{\mathbf {S}}_{\Omega }{\mathbf {m}}_{i}\right) \right) . \end{aligned}$$

Then the sought-after *optimal sampling pattern* of size *M* is:

3 $$\begin{aligned} {\hat{\Omega }}=\mathop {{\mathrm{arg\,min}}}\limits _{\underset{s.t.\,|\Omega |=M}{\Omega \subset \Gamma }}F(\Omega ). \end{aligned}$$

### Models used

Parallel MRI methods that directly reconstruct the images, such as sensitivity encoding method (SENSE)^16,75^ and many CS approaches^76^, are based on an image-to-k-space forward model, such as

4 $$\begin{aligned} {\mathbf {m}}=\mathbf {\mathbf {FC}x}=\mathbf {Ex}, \end{aligned}$$

where ${\mathbf {x}}$ is a vector that represents a 2D+time image of size $N_{y} \times N_{z} \times N_{t}$ ($N_{y}$ and $N_{z}$ are horizontal and vertical dimensions, $N_{t}$ is the number of time frames), ${\mathbf {C}}$ denotes the coil sensitivities transform mapping ${\mathbf {x}}$ into multi-coil-weighted images of size $N_{y} \times N_{z} \times N_{t} \times N_{c}$, with $N_{c}$ coils. ${\mathbf {F}}$ represents the spatial Fourier transforms (FT), comprising $N_{t} \times N_{c}$ repetitions of the 2D-FT, and ${\mathbf {m}}$ is the fully sampled measurements, of size $N_{y} \times N_{z} \times N_{t} \times N_{c}$. The two transforms combine into the encoding matrix ${\mathbf {E}}$. In 2D+time problems $N = N_{y}N_{z}N_{t}$ and $N_{s}=N_{c}$, while in 1D problems $N = N_{y}$, $N_{s}=N_{z}N_{c}$, and $N_{t}=1$. In this work, all vectors, such as ${\mathbf {m}}$ and ${\mathbf {x}}$, are represented by bold lowercase letters, and all matrices or linear operators, such as ${\mathbf {C}}$ or ${\mathbf {F}}$, are represented by bold uppercase letters.

When accelerated MRI by undersampling is used, the sampling pattern is included in the model as

5 $$\begin{aligned} \bar{{\mathbf {m}}}={\mathbf {S}}_{\Omega }\mathbf {\mathbf {FC}x}, \end{aligned}$$

where ${\mathbf {S}}_{\Omega }$ is the sampling function using SP $\Omega$ (same for all coils) and $\bar{{\mathbf {m}}}$ is the undersampled multi-coil k-space measurements (or k-t-space when $N_{t}>1$), with $N_{s}{M}$ elements, recalling that the AF is *N*/*M*. For reconstructions based on this model, we assumed that a central area of the k-space is fully sampled (such an area is used to compute coil sensitivities with auto-calibration methods, as in^77^).

In parallel MRI methods that recover the multi-coil k-space directly, the undersampling formulation is given by (1) and the image-to-k-space forward model is not used, since one is interested in recovering missing k-space samples using e.g. structured low-rank models^23^. For this, the multi-coil k-space is lifted into a matrix ${\mathbf {H}}=H({\mathbf {m}})$, assumed to be a low-rank structured matrix. Lifting operators $H({\mathbf {m}})$ are slightly different across PI methods, exploiting different kinds of low-rank structure^7–11,23^.

Once all the samples of the k-space are recovered, the image can be computed by any coil combination^78,79^, such as:

6 $$\begin{aligned}{}[\hat{{\mathbf {x}}}]_{n}={\textstyle \sum _{c=1}^{N_{c}}}{\mathbf {w}}_{n,c} [{\mathbf {F}}_{c}^{-1}{\mathbf {m}}_{c}]_{n}, \end{aligned}$$

where ${\mathbf {m}}_{c}$ is the measurements from coil *c*, ${\mathbf {F}}_{c}^{-1}$ is the inverse 2D-FT for one coil and ${\mathbf {w}}_{n,c}$ is the weight for spatial position *n* and coil *c*.

### Reconstruction methods tested

We tested our proposed approach on five different reconstruction methods: Three one-frame parallel MRI methods (SENSE^75^, P-LORAKS^11^, and PI-CS with anisotropic TV^80,81^) and two multi-frame low-rank and PI-CS methods for quantitative MRI^12^.

In P-LORAKS^11,82^ the recovery from $\bar{{\mathbf {m}}}$ produces:

7 $$\begin{aligned} R(\Omega ,\bar{{\mathbf {m}}})=\underset{\underset{s,t.\mathbf {S}_{\Omega } \mathbf {m}=\bar{{\mathbf {m}}}}{{\mathbf {m}}}}{{\mathrm{arg\,min}}}\left\| H_{s}({\mathbf {m}})-H_{s,r}({\mathbf {m}})\right\| _{F}^{2}, \end{aligned}$$

where the operator $H_{s}({\mathbf {m}})$ produces a low-rank matrix and $H_{s,r}({\mathbf {m}})$ produces a hard threshold version of the same matrix. P-LORAKS exploits consistency between the sampled k-space measurements and reconstructed measurements; it does not require a regularization parameter. Further, it does not need pre-computed coil sensitivities, nor fully sampled k-space areas for auto-calibration.

SENSE, CS, or low-rank (LR) reconstruction^12^ is given by:

8 $$\begin{aligned} \hat{{\mathbf {x}}}=\underset{{\mathbf {x}}}{{\mathrm{arg\,min}}}\left( \left\| \bar{{\mathbf {m}}}-{\mathbf {S}}_{\Omega }\mathbf {{\mathbf {E}}x}\right\| _{2}^{2}+\lambda P({\mathbf {x}})\right) \approx R_{{\mathbf {x}}}(\Omega ,\bar{{\mathbf {m}}}), \end{aligned}$$

where $\lambda$ is a regularization parameter. For SENSE, $\lambda =0$ and no regularization is used. For CS and LR, we looked at the regularizations: $P({\mathbf {x}})=\left\| \mathbf {Tx}\right\| _{1}$, with ${\mathbf {T}}$ the spatial finite differences (SFD); and low rank (LR), using nuclear-norm of ${\mathbf {x}}$ reordered as a Casorati matrix $P({\mathbf {x}})=\left\| {\mathbf {M}}({\mathbf {x}})\right\| _{*}$^83^.

CS approaches using redundant dictionaries ${\mathbf {D}}$ in the synthesis models^24,84^, given by ${\mathbf {x}}=\mathbf {Du}$, can be written as:

9 $$\begin{aligned} \hat{{\mathbf {x}}}=\mathbf {{\mathbf {D}}}\cdot \underset{{\mathbf {u}}}{{\mathrm{arg\,min}}}\left( \left\| \bar{{\mathbf {m}}}-{\mathbf {S}}_{\Omega }\mathbf {\mathbf {ED}u}\right\| _{2}^{2}+\lambda \left\| {\mathbf {u}}\right\| _{1}\right) \approx R_{{\mathbf {x}}}\left( \Omega ,\bar{{\mathbf {m}}}\right) . \end{aligned}$$

A dictionary to model exponential relaxation processes, like $\text {T}_{2}$ and $\text {T}_{1\rho }$, in MR relaxometry problems is the multi-exponential dictionary^12,85^. It generates a multicomponent relaxation decomposition^86^. The approximately-equal symbol $\approx$ is used in (8) and (9), since the iterative algorithm for producing $R_{{\mathbf {x}}}(\Omega ,\bar{{\mathbf {m}}})$, MFISTA-VA^87^ in this paper, may stop before reaching the minimum.

### Criteria utilized in this paper

We work primarily with a criterion defined in the multi-coil k-space; see (2) and (3). This criterion is used by parallel MRI methods that recover the k-space components directly in a k-space interpolation fashion (and not in the image-space), such as P-LORAKS^11^ and others^7,23,25^. Unless stated otherwise, the $f({\mathbf {m}},{\mathbf {n}})$ in (2) is

10 $$\begin{aligned} f({\mathbf {m}},{\mathbf {n}})=\dfrac{\left\| {\mathbf {m}}-{\mathbf {n}}\right\| _{2}^{2}}{\left\| {\mathbf {m}}\right\| _{2}^{2}}. \end{aligned}$$

The term $\left\| {\mathbf {m}}\right\| _{2}^{2}$ normalizes the error, so that the cost function will not be dominated by datasets with a strong signal.

For image-based reconstruction methods (e.g., SENSE and multi-coil CS) using the model in (4), the $R\left( \Omega ,{\mathbf {S}}_{\Omega }{\mathbf {m}}_{i}\right)$ in (2) is replaced by ${\mathbf {E}}R_{{\mathbf {x}}}\left( \Omega ,{\mathbf {S}}_{\Omega }{\mathbf {m}}_{i}\right)$, as defined, e.g.*,* in (8) and (9). The approach used to obtain the coil sensitivity is part of the method.

Note that (3) can be modified for image-domain criteria as well, such as:

11 $$\begin{aligned} {\hat{\Omega }}=\mathop {{\mathrm{arg\,min}}}\limits _{\underset{s.t.|\Omega |=M}{\Omega \subset \Gamma }} \left( \frac{1}{N_{i}}\sum _{i=1}^{N_{i}}g\left( {\mathbf {x}}_{i}, R_{{\mathbf {x}}}(\Omega ,{\mathbf {S}}_{\Omega }{\mathbf {m}}_{i})\right) \right) , \end{aligned}$$

where $g\left( {\mathbf {x}},{\mathbf {y}}\right)$ is a measurement of the distance between images ${\mathbf {x}}$ and ${\mathbf {y}}$. In this case, the fully-sampled reference must be computed using a reconstruction algorithm, such as ${\mathbf {x}}_{i}=R_{{\mathbf {x}}}(\Gamma ,{\mathbf {m}}_{i})$, and so it is dependent on to the parameters used in that algorithm.

### Proposed data-driven optimization

Due to the high computational cost of greedy approaches for large SPs and the relatively low cost of predicting points that are good next candidates, we propose a new learning approach, similar to POSS^64,66,67^, but with new heuristics that significantly accelerates the subset selection. For a general description of POSS see^64^, Algorithm 14.2.

**Figure 2:**
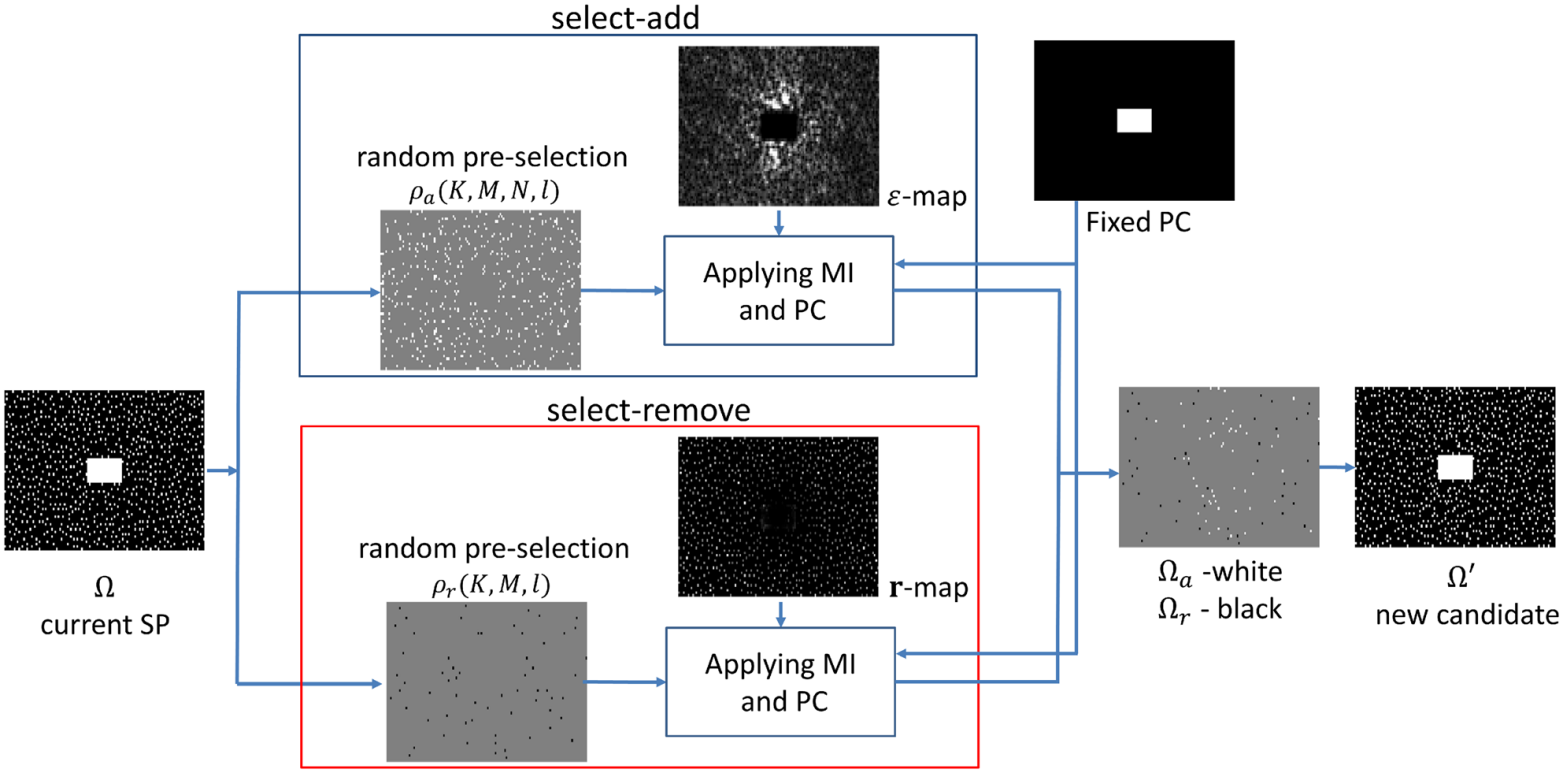
Illustration of the steps used in the functions** select-add** and **select-remove**. First, a random pre-selection is done by Bernoulli trials, using probabilities $\rho _{a}(K,M,N,l)$ and $\rho _{r}(K,M,l)$. Later selections are made based on the measurement of importance, using the $\varepsilon$-map and the ${\mathbf {r}}$-map (in which brighter indicates higher values), and the positional constraints (which include identification of the fixed areas). The resulting $\Omega _{a}$ is shown in white and $\Omega _{r}$ in black in the process of composing $\Omega '$. The new candidate $\Omega '$ is accepted if the cost function is reduced. These steps are repeated at each iteration.

In our proposed method, similarly to POSS, there is a sequential random selection of the elements to be changed. Differently from POSS, two heuristic rules, named the measure of importance (MI) and the positional constraints (PCs), are used to bias the selection of the elements with the intent to accelerate convergence. This is why our algorithm is named bias-accelerated subset selection (BASS). The MI (defined explicitly in (16) below) is a weight assigned to each element, indicating how much it is likely to contribute to decreasing the cost function. The PCs are rules for avoiding selecting undesirable elements, which may be one of two types: *fixed* or *relative*. Fixed positional constraints rule out the selection of an element because there is some prior reason for *fixing* its value (for example, elements used for auto-calibration are often considered to be such, an area of such elements is illustrated in Fig. 2, top right). Relative positional constraints are inspired by those used in the general combinatorial optimization approach called tabu search (TS)^88^, that had been demonstrated to be effective optimization approaches, in which a selection of some elements results in the forbidding of some otherwise legitimate selections in the same iteration. The rules that we have found efficacious in our application are that if an element with high MI is selected, then an adjacent element and also elements that are in complex-conjugated positions should not be selected in the same iteration. However, this does not prevent them to be selected in future iterations.

BASS, aims at finding (an approximation of) the ${\hat{\Omega }}$ of (3), is described in Algorithm 1. It uses the following user-defined items:

- $\Omega _{init}$ is the initial SP for the algorithm. It may be any SP (a Poisson disk, a variable density or even empty SP).
- *L* is the number of iterations in the training process.
- *N* is the number of positions in the fully-sampled set $\Gamma$.
- *M* is the desired size of the SP ($M<N$).
- $K_{init}$ is the maximum (initial) number of elements to be added/removed per iteration ($K_{init}<\min (M,N-M)$).
- $\rho _{r}$ is a function of three positive-integer variables *K*, *M*, where ($K<M$), and *l*, such that $K/M<\rho _{r}(K,M,l)\le 1.$
- $\rho _{a}$ is a function of the positive-integer variables *K*, *M*, *N*, where ($K<N-M$), and *l*, such that $K/(N-M)<\rho _{a}(K,M,N,l)\le 1$.
- **select-remove**$\left( \Omega ,K,\rho _{r}(K,M,l)\right)$ is a subset of $\Omega ,$ specified below.
- **select-add**$\left( \Omega ,K,\rho _{a}(K,M,N,l)\right)$ is a subset of $\Gamma \backslash \Omega ,$ specified below.
- *F* is an efficacy function; see (2) with the following.
  - $N_{i}$ is the number of items in the training set.
  - ${\mathbf {m}}_{1},\ldots ,{\mathbf {m}}_{N_{i}}$ are the measurements items in the training set.
  - *R* is the recovery algorithm from undersampled measurements.
- $\alpha$ is a reduction factor for the number of elements to be added/removed per iteration ($0<\alpha <1$).

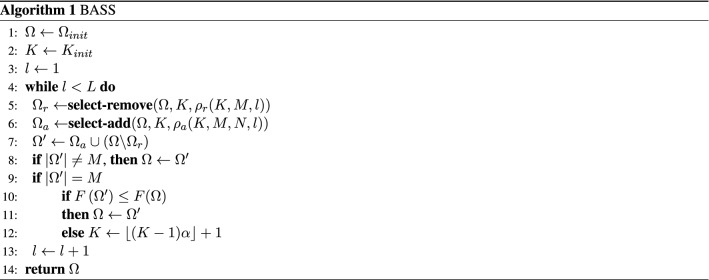

### Selection of elements to be added to or removed from the SP

Elements of $\Omega _{a}$ and $\Omega _{r}$ are selected by the functions **select-add** and **select-remove** in similar ways, described in the following paragraphs. First, we point out properties of those selections that ensure the progress of the learning algorithm toward finding an SP of *M* elements. The properties in question are that if $\Omega _{r}$, $\Omega _{a}$, and $\Omega '$ are obtained by Steps 5, 6 and 7 of Algorithm 1, then

12 $$\begin{aligned} \left| \Omega _{a}\right|= & {} \min \left( \max (M+K-|\Omega |,0),K\right) , \end{aligned}$$

13 $$\begin{aligned} \left| \Omega _{r}\right|= & {} \min \left( \max (|\Omega |+K-M,0),K\right) ,\end{aligned}$$

14 $$\begin{aligned} \left| \Omega '\right|= & {} \left| \Omega \right| +\left| \Omega _{a}\right| -\left| \Omega _{r} \right| . \end{aligned}$$

It follows that if $\left| \Omega \right| <M$, then $\left| \Omega _{r}\right| <\left| \Omega _{a}\right| =K$ and if $\left| \Omega \right| >M,$ then $\left| \Omega _{a}\right| <\left| \Omega _{r}\right| =K$. Consequently, if $\left| \Omega \right| \ne M$, then

15 $$\begin{aligned} \left| \left| \Omega '\right| -M\right| <\left| \left| \Omega \right| -M\right| . \end{aligned}$$

On the other hand, if $\left| \Omega \right| =M$, then $\left| \Omega '\right| =\left| \Omega \right|$. Thus, executing Algorithm 1 results in a sequence of $\left| \Omega \right|$ that converges to *M*.

We now define (and illustrate in Fig. 2) **select-add**$\left( \Omega ,K,\rho _{a}(K,M,N,l)\right)$ and **select-remove**$\left( \Omega ,K,\rho _{r}(K,M,l)\right)$ in Algorithm 1; they are used in steps 5, 6, and 7. Intuitively, the definitions should be such that the SP $\Omega '$ after step 7 is an improved choice as compared to the SP $\Omega$. The number *K* of elements to be added/removed varies with iteration.

For $1\le i\le N_{i}$, let ${\mathbf {e}}_{i}={\mathbf {m}}_{i}-R\left( \Omega ,{\mathbf {S}}_{\Omega }{\mathbf {m}}_{i}\right)$. Here, ${\mathbf {e}}_{i}$ is $N_{s}N$-dimensional vector comprised of *N* elements, indexed by $1\le k\le N$, each of which is an $N_{s}$-dimensional vector, with components indexed by $1\le s\le N_{s}$; we use $[{\mathbf {e}}_{i}]_{k,s}$ to denote the *s*th component of the *k*th component of ${\mathbf {e}}_{i}$. The *k*th component of ${\mathbf {e}}_{i}$ comprises the $N_{s}$ measurements related to the *k*th component of the SP.

For **select-add**, we define a measure of importance (MI) used in this work, for $1\le k\le N$, as

16 $$\begin{aligned} \varvec{\varepsilon }{}_{k}=\frac{1}{N_{i}N_{s}} \sum _{i=1}^{N_{i}}\frac{\sum _{s=1}^{N_{s}} \left| [{\mathbf {e}}_{i}]_{k,s}\right| ^{2}}{\left\| {\mathbf {m}}_{i}\right\| _{2}^{2}}, \end{aligned}$$

referred to as the $\varvec{\varepsilon }$-map. The purpose of **select-add** is to select *K* elements from $\Gamma \backslash \Omega$ in the following randomly-biased manner. First, an approximately $\rho _{a}\times (N-M)$ number of elements are randomly pre-selected by Bernoulli trials with $\rho _{a}$ probability, whose value is the user-provided $\rho _{a}(K,M,N,l)$ (recall that $K/(N-M)<\rho _{a}(K,M,N,l)\le 1$). To have more than *K* pre-selected points, we need $\rho _{a}>K/(N-M)$. The *K* best points of the random pre-selected points will be chosen. The selection starts sequentially with the element with largest MI (the largest $\varepsilon _{k}$). Once this element is chosen, any other element identified as undesirable by the PC rules is excluded from the randomly pre-selected group, and the selection continues with the element with next largest MI. The chosen *K* elements are likely to be useful for the aim of (3). The probability $\rho _{a}$ indirectly controls the bias applied to the selected set. Larger probability implies less randomness and more bias. The probability varies with iteration *l*.

For **select-remove,** a sequence with number of elements specified in (13), that are in $\Omega$, is generated in the same way, but using ${\mathbf {r}}{}_{k}$ as the MI, instead of $\varepsilon _{k}$:

17 $$\begin{aligned} {\mathbf {r}}{}_{k}=\frac{1}{N_{i}N_{s}}\sum _{i=1}^{N_{i}} \frac{\sum _{s=1}^{N_{s}}\left| [{\mathbf {e}}_{i}]_{k,s}\right| ^{2} +\delta }{\sum _{s=1}^{N_{s}}\left| [{\mathbf {m}}_{i}]_{k,s}\right| ^{2}+\delta }, \end{aligned}$$

for $1\le k\le N$ with $\delta$ a small constant to avoid zero/infinity in the defining of ${\mathbf {r}}{}_{k}$, which is referred to as the ${\mathbf {r}}$-map. The idea of this MI is that a large reconstruction error in a sampled k-space position *k*, defined as $\sum _{s=1}^{N_{s}}\left| [{\mathbf {e}}_{i}]_{k,s}\right| ^{2}$, where the expected quadratic value of the element is relatively small, defined as $\sum _{s=1}^{N_{s}}\left| [{\mathbf {m}}_{i}]_{k,s}\right| ^{2}$, renders that element as less important for the SP. The elements of this sequence comprise $\Omega _{r}$, to be removed from $\Omega$ in the process of composing $\Omega '$.

The probability of pre-selecting elements should respect pre-defined ranges, $K/M<\rho _{r}(K,M,l)\le 1$ and $K/(N-M)<\rho _{a}(K,M,N,l)\le 1$. In order to take advantage of the convergence properties of POSS^64^, we argue that the probabilities should be reduced along iterations. In later iterations, according to line 12 of Algorithm 1, we have $K\rightarrow 1$ (when $K=1$, the relative PC are no longer used), then we should also have $\rho _{r}(K,M,l)\rightarrow 1/M$ and $\rho _{a}(K,M,N,l)\rightarrow 1/(N-M)$ for BASS to become like POSS, when the change of elements are purely random. At this point, the same convergence properties of POSS, stated in^64^ applies to BASS, given that monotonicity and submodularity are valid. Our recommended choices for lines 6 and 5 of Algorithm 1 are

18 $$\begin{aligned} \rho _{r}(K,M,l)=K/M+((M-K)/(M\times l)), \end{aligned}$$

and

19 $$\begin{aligned} \rho _{a}(K,M,N,l)=K/(N-M)+((N-M-K)/((N-M)\times l)), \end{aligned}$$

with *l* the iteration index. This results in more bias in the beginning of the iterative process and more randomness in later iterations. Other rules can be used, even constant probabilities ($\rho _{r}(K,M,l)>1/M$ and $\rho _{a}(K,M,N,l)>1/(N-M)$) for a specific number of iterations. The same PCs were used in **select-add** and **select-remove**.

The expensive part of **select-add** and **select-remove** is the computation of the recoveries given by $R\left( \Omega ,{\mathbf {S}}_{\Omega }{\mathbf {m}}_{i}\right)$, but this is done only once per iteration, for all $N_{i}$ images. These recoveries are also reused to calculate the cost *F* in line 10 of Algorithm 1. Figure 2 illustrates the steps of these functions using $K=50$.

## Methods

### Datasets

In our experiments we utilized three datasets. One dataset, denominated $\text {T}_{2}$-*brain*, contains 40 brain $\text {T}_{2}$-weighted images and k-space measurements from the fast MRI dataset of^89^. Of these, $N_{i}=30$ were used for training and $N_{v}=10$ for validation. The k-space measurements are of size $N_{y} \times N_{z} \times N_{t} \times N_{c}=320 \times 320 \times 1 \times 16$, and the reconstructed images are $N_{y} \times N_{z} \times N_{t}=320 \times 320 \times 1$. With this dataset, we tested 2D SPs, of size $N=320 \times 320$, and 1D SPs, of size $N=320$ (see Fig. 1). We used 1D SPs with experiments with large numbers of iterations to compare BASS against POSS and greedy approaches. The fast MRI dataset is a public dataset composed of images and k-space data obtained with different acquisitions, not all of them are 3D acquisitions. In this sense, the experiments with 2D SPs in the $\text {T}_{2}$-*brain* dataset are merely illustrative.

The second dataset, $\text {T}_{1\rho }$-*brain*, contains $\text {T}_{1\rho }$-weighted k-space measurements of the brain, of size $N_{y} \times N_{z} \times N_{t} \times N_{c}=128 \times 148 \times 1 \times 20$, and the reconstructed images are $N_{y} \times N_{z} \times N_{t}=128 \times 148 \times 1$. Unless otherwise stated, $N_{i}=65$ were used for training and $N_{v}=16$ for validation. This dataset and the next one were all acquired with the Cartesian 3D acquisitions as described in “The specific content of this paper”, training and validation sets are composed of data from different individuals.

The third dataset, denominated $\text {T}_{1\rho }$-*knee*, contains $\text {T}_{1\rho }$-weighted knee images and k-space measurements for quantitative $\text {T}_{1\rho }$ mapping, of size $N_{y} \times N_{z} \times N_{t} \times N_{c}=128 \times 64 \times 10 \times 15$, and the reconstructed images $N_{y} \times N_{z} \times N_{t}=128 \times 64 \times 10$ representing the cross-sections of the human knee, and 2D+time SPs of size $N=128 \times 64 \times 10$. Unless otherwise stated, $N_{i}=30$ were used for training and $N_{v}=10$ for validation. The k-space measurements for all images are normalized by the largest component. A *reduced-size knee* dataset uses only part of the $\text {T}_{1\rho }$-knee dataset. Images are of size $N_{y} \times N_{z} \times N_{t}=128 \times 64 \times 1,$ and $N_{i}=5$ and $N_{v}=5$. This dataset is used in experiments with a large number of iterations to compare BASS against POSS and greedy approaches for 2D SPs.

### Reconstruction methods

For the $\text {T}_{2}$-brain and $\text {T}_{1\rho }$-brain datasets, three reconstruction methods were used:

- SENSE^75^: Multi-coil reconstruction, following Eq. (8) with $\lambda =0$, and minimized with conjugate gradient.
- P-LORAKS^11^: from Eq. (7), with codes available online (https://mr.usc.edu/download/loraks2/).
- CS-SFD^87^: Multi-coil CS with sparsity in the spatial finite differences (SFD) domain, following Eq. (8), and minimized with MFISTA-VA.

SENSE was used only for 1D SP comparisons between BASS, POSS and greedy approaches.

For the $\text {T}_{1\rho }$-weighted knee dataset, we used different methods:

- CS-LR^12^: Multi-coil CS using nuclear-norm of the vector ${\mathbf {x}}$ reordered as a Casorati matrix $P({\mathbf {x}})=\left\| {\mathbf {M}}({\mathbf {x}})\right\| _{*}$ and minimized with MFISTA-VA.
- CS-DIC^12^: Multi-coil CS using synthesis approach following Eq. (9), using ${\mathbf {D}}$ as a multi-exponential dictionary^85^, and minimized with MFISTA-VA.

CS-SFD, CS-LR, and CS-DIC need a fully-sampled area for auto-calibration of coil sensitivities using ESPIRiT^77^. P-LORAKS does not use auto-calibration. All experiments were performed in Matlab, codes used in this manuscript are available in https://cai2r.net/resources/software/data-driven-learning-sampling-pattern.

The regularization parameter (the $\lambda$ in (8) and (9)) required in CS-SFD, CS-LR, and CS-DIC was optimized independently for each type of SP (Poisson disk, variable density, combined variable density and Poisson disk, adaptive SP, or optimized) and each AF, using the same training data. The parameters of the recovery method *R* are assumed to be fixed during the learning process of the SP.

### Optimizing parameters of Poisson disk, variable density, and adaptive SPs

Grid optimization with 50 realizations of each SP was performed, changing the parameters used to generate these SPs, to obtain the best realization of these SPs, which corresponds to the one that minimizes $F(\Omega )$. This approach is the one used in^56^ for minimizing $F(\Omega )$. Poisson disk and variable density codes used in the experiments are at https://github.com/mohakpatel/Poisson-Disc-Sampling and http://mrsrl.stanford.edu/~jycheng/software.html. Combined Poisson disk and variable density SP from^44^ and adaptive SPs from^45^ were also used for comparison. The spectrum template obtained from the same training data was used with adaptive SPs. All these approaches can be considered data-driven approaches because optimization of the parameters to generate the SP was performed. They all have a fixed computational cost of $50N_{i}$ image reconstructions (nearly the same computational cost as BASS).

### Evaluation of the error

The quality of the results obtained with the SP was evaluated using the normalized root mean squared error (NRMSE):

20 $$\begin{aligned} \text {NRMSE}\left( \left\{ {\mathbf {m}}_{i}\right\} _{i=1}^{N_{v}},\left\{ \hat{{\mathbf {m}}}_{i}\right\} _{i=1}^{N_{v}}\right) =\sqrt{{\textstyle \sum _{i=1}^{N_{v}}\frac{\left\| {\mathbf {m}}_{i}-\hat{{\mathbf {m}}}_{i}\right\| _{2}^{2}}{\left\| {\mathbf {m}}_{i}\right\| _{2}^{2}}}}. \end{aligned}$$

When not specified, the NRMSE shown was obtained from k-space on the validation set; results using image-domain and the training set are also provided, as is structural similarity (SSIM)^90^ in some cases.

## Results

### Illustration of the convergence and choice of parameters

**Figure 3:**
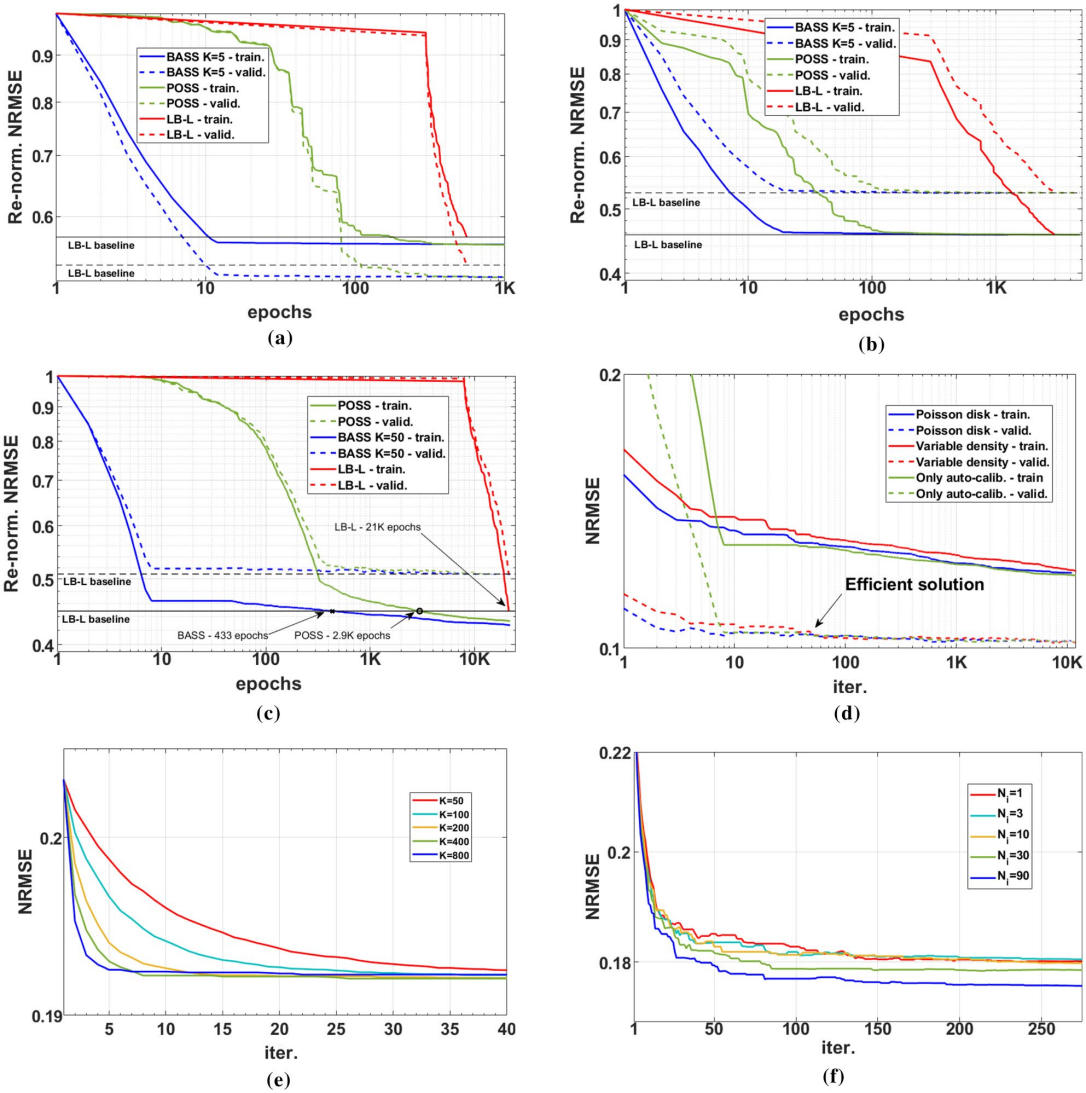
Convergence curves for BASS. Comparison against POSS and the greedy approach LB-L in (**a**) 1D SP using SENSE, (**b**) 1D SP using CS-SFD, and (**c**) 2D SP using CS-SFD. (**d**) Comparing various initial SPs. (**e**) Comparing various $K_{init}$s. (**f**) Comparing various training sizes.

In Fig. 3a–c we compare BASS against POSS^66^ and the greedy approach “learning-based lazy” (LB-L)^56^, adapted to the cost function in (2). The resulting NRMSEs are re-normalized by the initial values and show the difference in computational cost and quality between the approaches. Plots are scaled logarithmically in epochs (in each “epoch” all the images are reconstructed once). In Fig. 3a, it is shown the performance of the learning methods with 1D SP using $\text {T}_{2}$*-brain* dataset and SENSE reconstruction, with AF = 4. In this example, BASS was faster than POSS and LB-L. Also, BASS and POSS obtained nearly same quality results, superior to LB-L. In Fig. 3b, the performance of the learning methods was tested in the same setup, but using CS-SFD reconstruction, with AF = 4. In this example, BASS was faster than POSS and LB-L, but all methods obtained nearly the same quality results. In Fig. 3c, the methods were compared with CS-SFD with the *reduced-size knee* dataset and 2D SPs, starting with the auto-calibration area and AF = 15. In this example BASS found a solution with same quality in the training set using only 433 epochs, around 50 times faster than LB-L (~ 21,000 epochs). Also, BASS and POSS can go on minimizing the cost function beyond the stopping point of LB-L finding even better SPs.

Figure 3d, demonstrates the performance of BASS for various initial SPs (same experimental setup as for Fig. 3c, with $K_{init}$=50). The improvement observable in the validation set ends quickly, at iteration 50 in this example. There is an arrow in the figure pointing to an efficient solution. Such a solution is obtained after relatively few iterations, during which most of the significant improvement observable with validation data has already happened. Iterating beyond this point essentially leads to marginal improvement, observable only with the training data.

In Fig. 3e we see the results of the learning process for the training data according to the parameters $K_{init}$ for CS-LR, AF = 20, using the* knee* dataset, with $N_{i}=30$ and $N_{v}=10$. Note that large $K_{init}$ performs better than small $K_{init}$ in terms of speed of convergence in the beginning of the learning process. Over time, *K* reduces from $K_{init}$ towards $K=1$.

The importance of large and diverse datasets to generate the learned sampling pattern for the specific class of images is illustrated in Fig. 3f, showing the convergence of the learning process with the validation set, in NRMSE. We used training sets of 1, 3, 10, 30, and 90 images. The validation sets were composed of the same 20 images, not used in any of the training sets.

**Figure 4:**
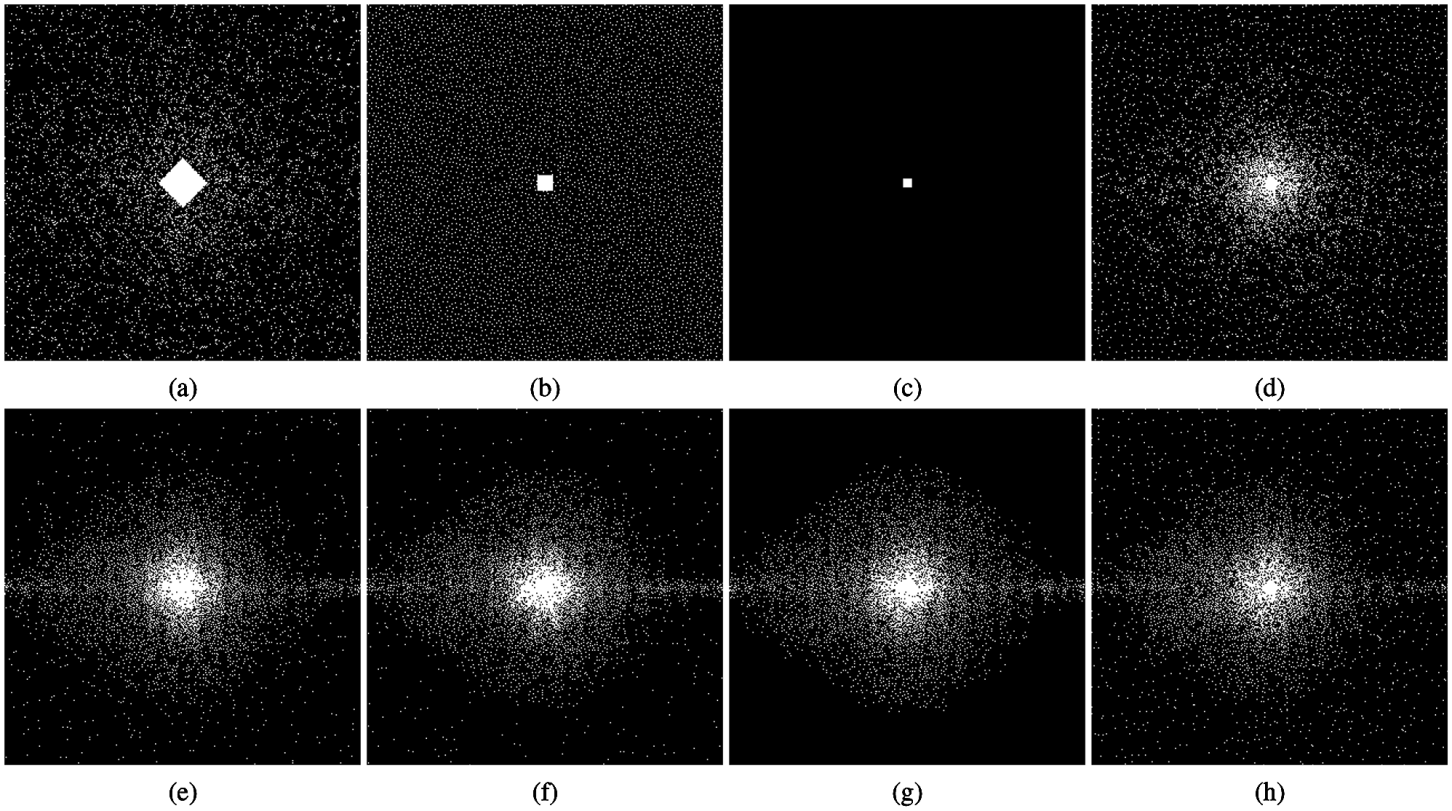
Efficient solutions produced for P-LORAKS with AF = 16 and initial SPs (**a**) variable density (VD), (**b**) Poisson disk (PD), (**c**) an SP that is empty except for a small central area (CA), and (**d**) adaptive SP. The corresponding efficient solutions are the SPs in (**e**) for VD ($\hbox {NRMSE}=0.196$), in (**f**) for PD ($\hbox {NRMSE}=0.195$), in (g) for CA ($\hbox {NRMSE}=0.194$), and in (**h**) for adaptive SP ($\hbox {NRMSE}=0.199$).

The robustness of an efficient solution in the presence of variable initial SP is illustrated in Fig. 4. Figure 4a–d show three initial SPs: variable density (VD), Poisson disk (PD), empty except for a small central area (CA), and adaptive SP. Using 200 iterations of BASS for P-LORAKS with these initial SPs, corresponding efficient SPs were obtained; shown in Fig. 4e–h. There are minor differences among them (around 1% difference in NRMSE), but the central parts of the SPs are very similar.

### Performance with various reconstruction methods

**Figure 5:**
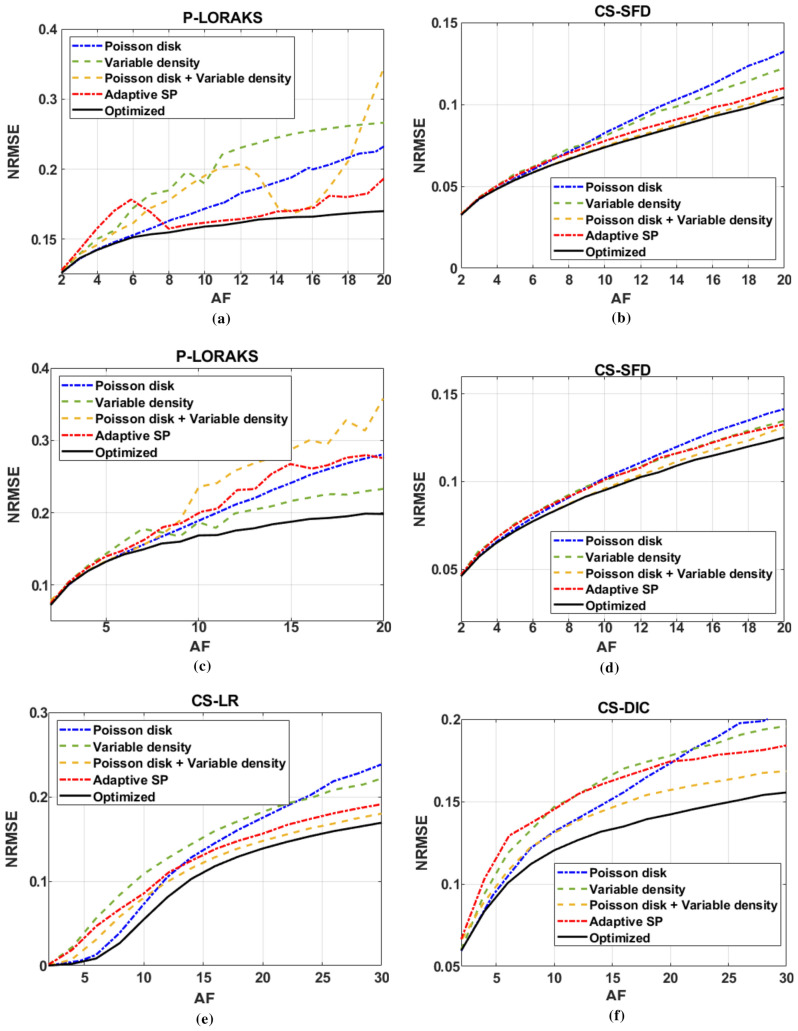
NRMSE (lower is better): (**a**) for P-LORAKS and (**b**) CS-SFD for $\text {T}_{2}$-*brain* dataset, (**c**) for P-LORAKS and (**d**) CS-SFD for $\text {T}_{1\rho }$-*brain*, dataset, and (**e**) for CS-LR, and (**f**) CS-DIC for $\text {T}_{1\rho }$-*knee* dataset. Variable density, Poisson disk, adaptive SP, and combined variable density with Poisson disk are compared with optimized SP (obtained by BASS) for various AFs.

BASS improves NRMSE in image space for fixed AFs when compared with the other SPs for the four reconstruction methods tested with 2D+time SPs. Figure 5a shows the NRMSE obtained by P-LORAKS with $\text {T}_{2}$-brain dataset, comparing variable density SPs, Poisson disk SPs, adaptive SPs, combined variable density with Poisson disk SPs, and the optimized SPs. Figure 5b shows the NRMSE obtained by CS-SFD with $\text {T}_{2}$-brain dataset. Figure 5c,d show P-LORAKS and CS-SFD with $\text {T}_{1\rho }$-brain, dataset. Figure 5e shows the NRMSE obtained by CS-LR with $\text {T}_{1\rho }$-knee dataset. Figure 5f shows the NRMSE obtained by CS-DIC with $\text {T}_{1\rho }$-knee dataset. All SP had their parameters optimized for each reconstruction method, dataset, and AF.

**Figure 6:**
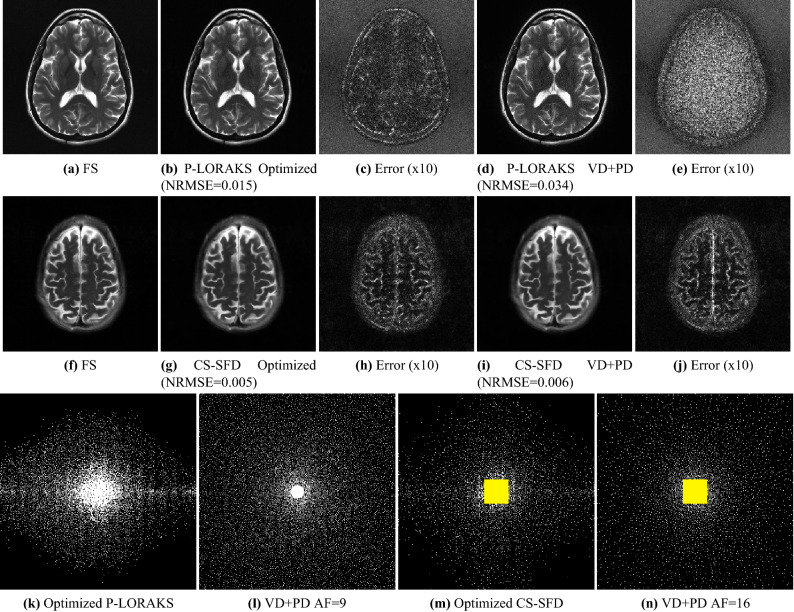
Visual results of the $\text {T}_{2}$-brain dataset are shown in this figure. For P-LORAKS, fully sampled (FS) reference is shown in (**a**), and images using optimized SP and combined variable density and Poisson disk (VD+PD) SP with AF = 9 are shown in (**b**) and (**d**). Error maps between FS and P-LORAKS are shown in (**c**) for Optimized SP and (**e**) for VD + PD SP. For CS-SFD, FS reference is shown in (**f**), and images using optimized SP and VD + PD SP with AF = 16 are shown in (**g**) and (**i**). Error maps between FS and CS-SFD are shown in (**h**) for Optimized SP and (**j**) for VD+PD SP. The optimized and VD+PD SPs with $\hbox {AF}=9$ for P-LORAKS are shown in (**k**) and (**l**), while the ones with $\hbox {AF}=16$ used for CS-SFD, with the highlighted central-square auto-calibration region, are shown in (**m**) and (**n**).

**Figure 7:**
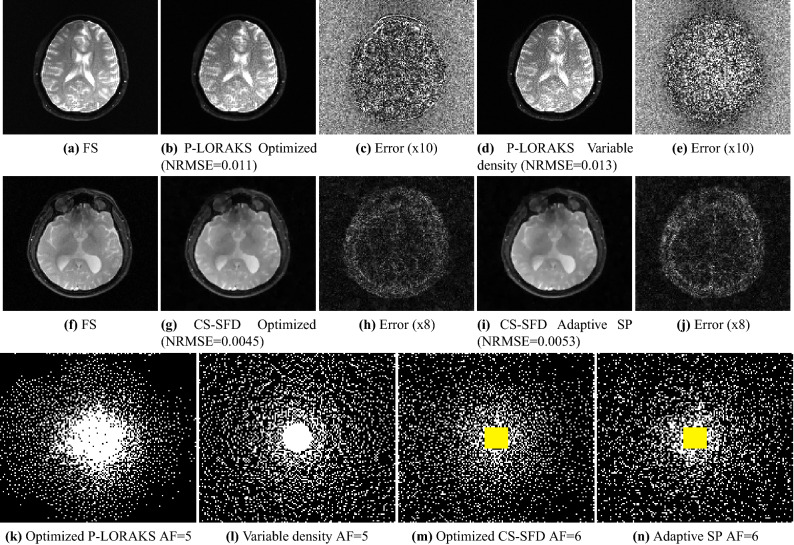
Visual results of the $\text {T}_{1\rho }$-brain dataset are shown in this figure. For P-LORAKS, fully sampled (FS) reference is shown in (**a**), and images using optimized SP and variable density SP with $\hbox {AF}=5$ are shown in (**b**) and (**d**). Error maps between FS and P-LORAKS are shown in (**c**) for Optimized SP and (**e**) for variable density SP. For CS-SFD, FS reference is shown in (**f**), and images using optimized SP and adaptive SP with $\hbox {AF}=6$ are shown in (**g**) and (**i**). Error maps between FS and CS-SFD are shown in (**h**) for Optimized SP and (**j**) for adaptive SP. The optimized and variable density SP with $\hbox {AF}=5$ for P-LORAKS are shown in (**k**) and (**l**), while the optimized and adaptive SPs with $\hbox {AF}=6$ used for CS-SFD, with the highlighted central-square auto-calibration region, are shown in (**m**) and (**n**).

Figure 6 illustrates on the $\text {T}_{2}$-brain dataset how the optimized SPs improve the reconstructed images with P-LORAKS (for AF = 9) and CS-SFD (for AF = 16) against combined variable density and Poisson disk (VD + PD). The P-LORAKS methods had a visible improvement in SNR, the CS-SFD methods became less smooth with some structures more detailed. However, some structured error can still be seen in the error maps. Figure 6 also illustrates that optimized SPs are different for the two reconstruction methods, even when using the same images for training. Figure 7 illustrates on the images obtained with the $\text {T}_{1\rho }$-brain dataset with P-LORAKS (for AF = 5) and CS-SFD (for AF = 6), comparing optimized SP with variable density and adaptive SP.

**Figure 8:**
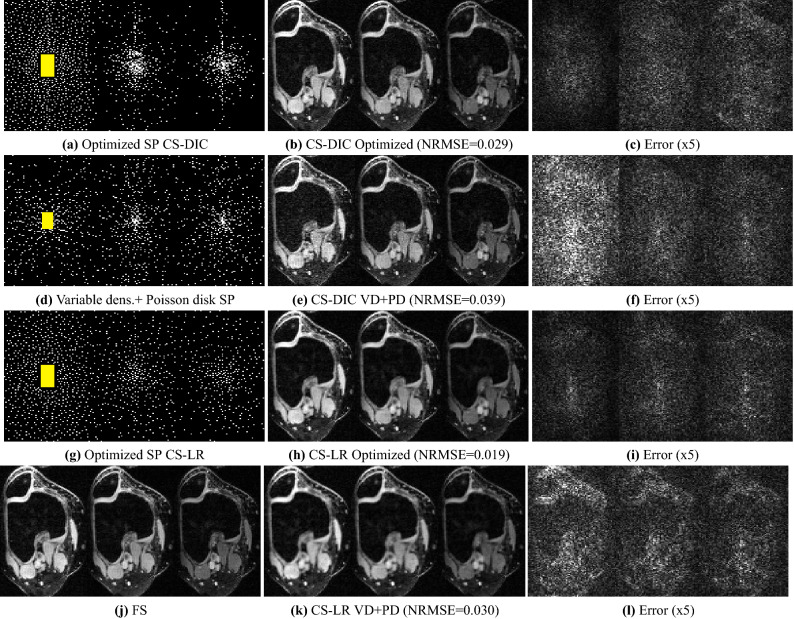
Three frames for different relaxation times of the knee dataset, when $\hbox {AF}=18$ was used, reconstructed with CS-DIC (**b**) and (**e**) and with CS-LR (**h**) and (**k**): compare these with the corresponding fully sampled (FS) measurements in (**j**), where the corresponding magnitude of the errors are in (**c,f,i,l**). Combined variable density and Poisson disk SP (**d**) and BASS optimized SPs for CS-DIC (**a**) and CS-LR (**g**) are also shown. Central auto-calibration area is highlighted in yellow.

In Fig. 8, visual results with the $\text {T}_{1\rho }$-knee dataset illustrate the improvement due to using an optimized SP as compared to using combined variable density and Poisson disk SP, for both CS-LR and CS-DIC. We also see that the optimized SPs are different for the two reconstruction methods. Note that both optimized k-t-space SPs have a different sampling density over time (first, middle, and last time frames are shown), being more densely sampled at the beginning of the relaxation process. The auto-calibration region is in the first frame.

### BASS with a different criterion

**Figure 9:**
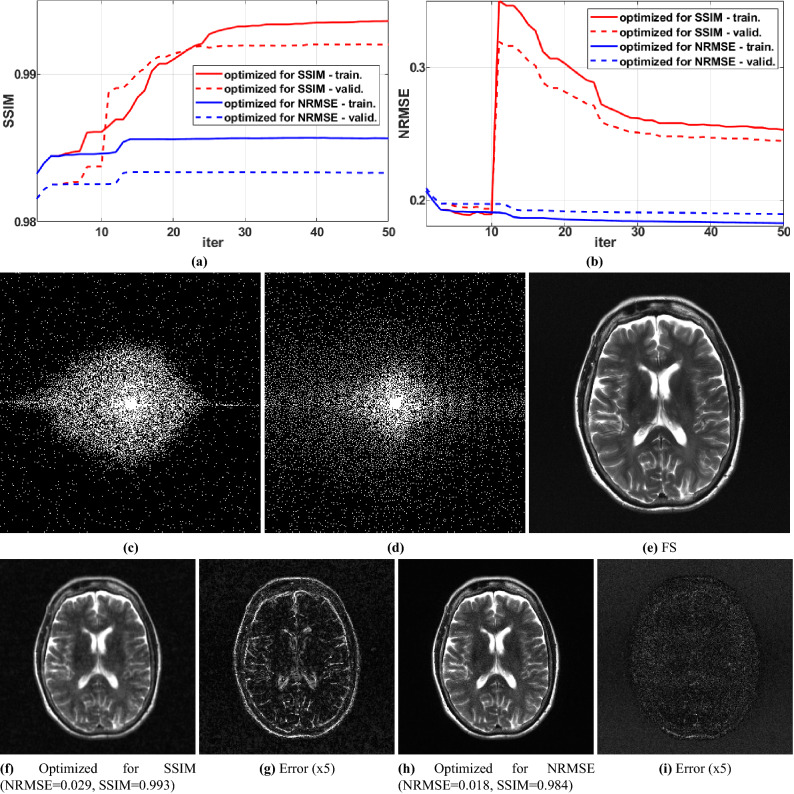
Comparing BASS in optimizing SSIM (higher is better) and NRMSE (lower is better). (**a**) SSIM and (**b**) NRMSE along the iterations, (**c**) SP obtained by optimizing SSIM, (**d**) SP obtained by optimizing NRMSE, and some visual results with fully sampled reconstruction in (**e**), example of images with SP obtained by optimizing SSIM in (**f**) and NRMSE in (**h**), and their error maps (**g**) and (**i**).

We illustrate that our proposed optimization approach is also efficacious with different criteria. In some applications, one may desire the best possible image quality, regardless of k-space measurements. Here we discuss the use of BASS to optimize the SSIM of^90^, an image-domain criterion. For that, the task in (3) of finding the minimizer of $F(\Omega )$ in (2), used in line 10 of the Algorithm 1, is replaced by finding the minimizer in (11), with $g\left( {\mathbf {x}},{\mathbf {y}}\right)$ the negative of the SSIM. In Fig. 9 we compare the optimization of SSIM with that of NRMSE, using P-LORAKS on the $\text {T}_{2}$-*brain* dataset, AF = 10, starting with the Poisson disk SP.

### $\text {T}_{1\rho }$ mapping

**Figure 10:**
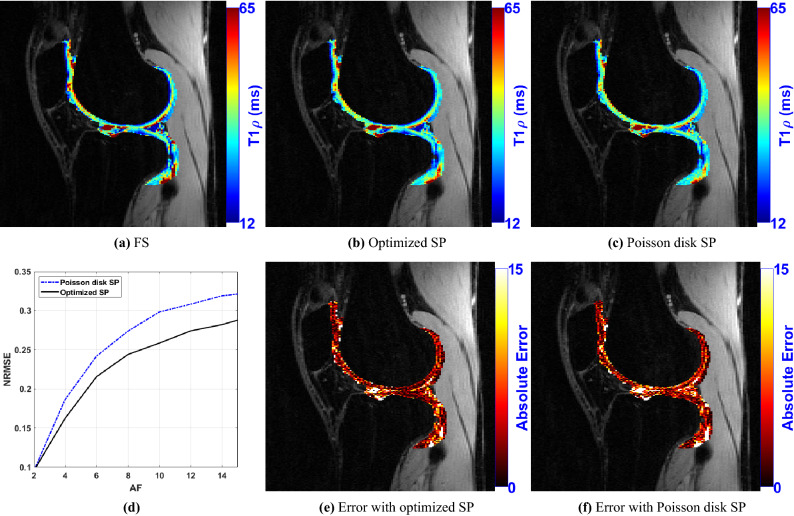
Comparison of $\text {T}_{1\rho }$ maps obtained with Poisson disk and optimized SP. Illustrative $\text {T}_{1\rho }$ maps obtained with fully sampled (FS) parallel MRI in (**a**), with optimized SP with $\hbox {AF}=4$ in (**b**), and with Poisson disk SP with $\hbox {AF}=4$ in (**c**). In (**d**), the NRMSE for different AF is shown. The point-wise error map between the $\text {T}_{1\rho }$ maps obtained between FS and optimized SP are shown in (**e**), and between FS and Poisson disk SP are shown in (**f**).

We illustrate the performance of the optimized SPs for $\text {T}_{1\rho }$ mapping. We compare the optimized SP against Poisson disk SP, previously used in^12^, for CS-LR. The SP and reconstructed images correspond to the cross-section of the knee, of size $N_{y} \times N_{z} \times N_{t}=128 \times 64 \times 10$, the $\text {T}_{1\rho }$ mapping is performed in the cartilage region on the longitudinal plane (in-plane) of the recovered 3D volume. The 3D+time volume has $N_{x} \times N_{y} \times N_{z} \times N_{t}=256 \times 128 \times 64 \times 10$ voxels, where $N_{x}=256$ corresponds to the samples in the frequency-encoding direction, field-of-view of $130\,\text{mm} \times 130\,\text{mm} \times 130\,\text{mm}$, and in-plane (rounded) resolution of $0.5\,\text{mm} \times 1\,\text{mm}$, slice thickness of $2\,{\text{mm}}$, and 10 frames. In Fig. 10 we illustrate the results with $\text {T}_{1\rho }$ mapping in the knee, particularly around the cartilage region. In Fig. 10a–c we show some illustrative $\text {T}_{1\rho }$ maps. In Fig. 10d we show the NRMSE for different acceleration factors, considering 10 slices containing the knee cartilage. In Fig. 10e,f, we show the point-wise errors of the $\text {T}_{1\rho }$ maps.

### Prospective accelerated scans

**Figure 11:**
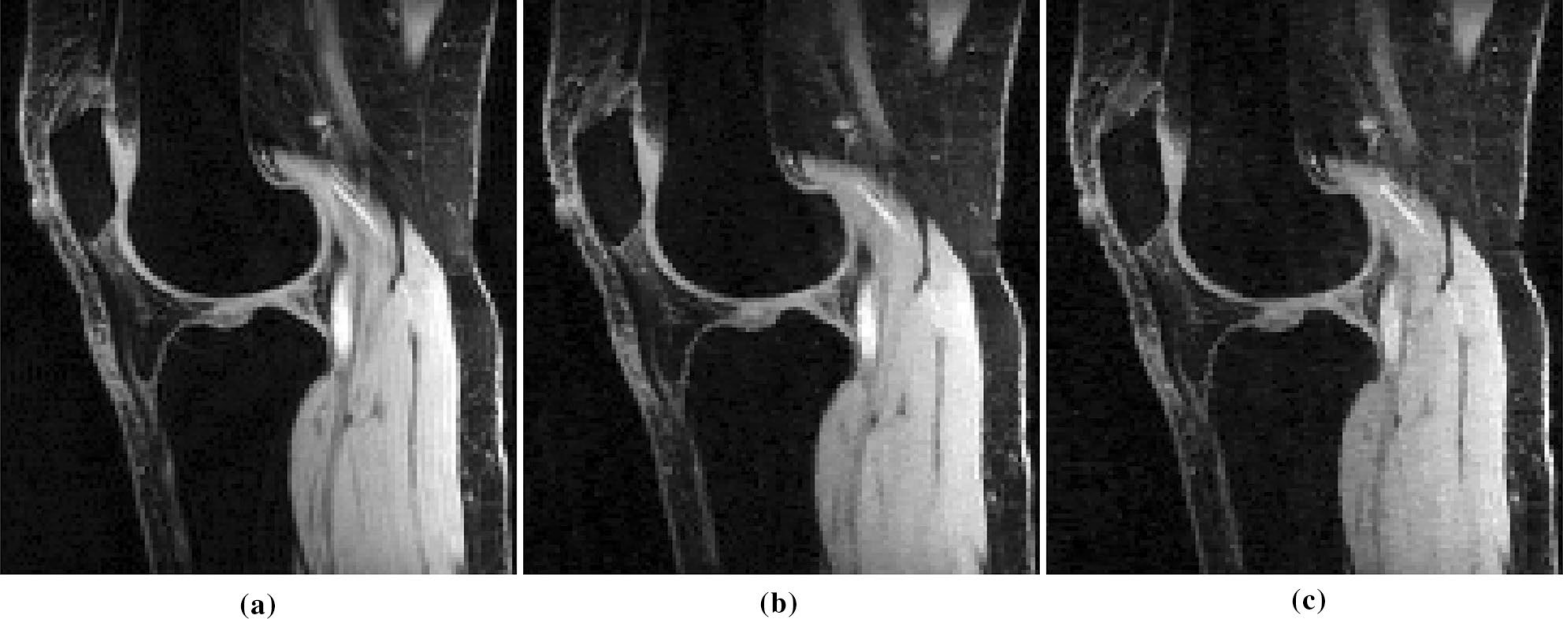
Comparison of (**a**) fully sampled parallel MRI (2 min 46 s scan time) with prospective accelerated parallel MRI at $\hbox {AF}=4$ (42 s scan time) using (**b**) optimized SP and (**c**) Poisson disk SP.

We tested the optimized SP obtained with BASS in prospective CS scans, in Fig. 11. For an explanation of the usage of the word “prospective” in MRI, see^6^. We used the knee dataset for training the SP for CS-SFD at AF = 4. The images of size $N_{y} \times N_{z} \times N_{t}=128 \times 64 \times 1$ used for training compose the cross-session of the 3D volumes. Displayed images correspond to the longitudinal view of one slice of a 3D volume (which has size $N_{x} \times N_{y} \times N_{z}=256 \times 128 \times 64$), with in-plane resolution of $0.5\,\text{mm} \times \text {1}\,\text{mm}$ and slice thickness of $2\,{\text{mm}}$. The 15-channel coil measurements was obtained with the $\text {T}_{1\rho }$ pulse sequence used in^12^, which is a $\text {T}_{1\rho }$ magnetization prepared fast gradient-echo sequence^2^.

## Discussion

The proposed approach delivers efficacious sampling patterns for high-resolution or quantitative parallel MRI problems. Compared to previous greedy approaches for parallel MRI, as in^56,57^, BASS is able to optimize much larger SPs, using larger datasets, spending less computational time than greedy approaches (Fig. 3a–c. Greedy approaches test considerably more candidates SPs before updating the SP. They are computationally affordable only for 1D undersampling or small 2D SPs, but they were inferior to BASS in computational time and imaging quality. Note that computational time for each epoch (or iteration for BASS) depends on the time of the implemented reconstruction algorithm. For CS-DIC, the reconstruction of each slice of the knee dataset takes 16.1 s on an NVIDIA Tesla V100 GPU, while for CS-LR it takes 9.6 s. For the $\text {T}_{2}$-brain dataset, P-LORAKS takes 110.2 s on a CPU Intel Skylake 2.4 GHz, SENSE takes 0.5 s and CS-SFD takes 6.3 s on an NVIDIA Tesla V100 GPU. We estimate approximate 1.5 computational years running LB-L (estimated 263 K epochs for learning) for 2D SP with $N=320 \times 320$ using CS-SFD on the $\text {T}_{2}$-brain dataset (considering only 30 training images) to obtain nearly the same SP that BASS finds in 33 hours (if 500 epochs or iterations are used) in an NVIDIA GPU.

The proposed approach for subset selection is effective because it uses a smart selection of new elements in the SP updating process. Candidates that are most likely to reduce the cost function are tried first. The obtained efficient solution may have minor differences depending on the initial SP (Fig. 4), but the optimized SPs tend to have the same final quality if more iterations are used (Fig. 3d). Adding and removing multiple elements at each iteration is beneficial for fast convergence at the initial iterations (Fig. 3e).

The cost function in (2) evaluates the error in k-space, not in the image domain. This may not be sufficiently flexible because it does not allow the specification of regions of interest in the image domain. Nevertheless, improvements measured by the image-domain criteria NRMSE were observed (Fig. 5). In different MRI applications other criteria than (2) may be desired. The proposed algorithm can be used for other criteria, such as the SSIM (Fig. 9).

The optimized SP varies with the reconstruction method (Figs. 6, 7 and 8) or with the optimization criterion (Fig. 9): thus sampling and reconstruction should be matched. This concept of matched sampling-reconstruction indicates that comparing different reconstruction methods with the same SP is not a fair approach, instead each MRI reconstruction method should be compared using its best possible SP. Note that the optimized SP improved the NRMSE by up to 45% in some cases (Fig. 5).

The experiments also show that optimizing the SP is more important at higher AFs. As seen in Fig. 5, the optimization of the SP flattened the curves of the error over AF, achieving a lower error with the same AF. For example, P-LORAKS with optimized SP at AF = 20 obtained the same level of NRMSE as with variable density SP at AF = 6, while CS-LR with optimized SP at AF = 30 obtained the same level as with Poisson disk SP at AF = 16, even after optimizing the parameters used to generate the Poisson disk SP. Thus it is possible to double the AF by optimizing the SP. Variable sampling rate over time is advantageous for $\text {T}_{1\rho }$ mapping as seen in^91^; it is interesting that the algorithm learned this, as shown in Figs. 8 and 10. It is also important to clarify that the results shown for variable density, Poisson disk, combined variable density and Poisson disk, and adaptive SP are the best obtained among a parameter optimization process spending 50 epochs. If a simple guess of the parameters for these SPs is used, then the performance of these SPs can be poor. In contrast, BASS found efficient SPs spending the same computational cost or less than that (10$\sim$50 epochs in Fig. 3a–d).

The lower computational cost and rapid convergence speed of BASS bring the advantage of learning the optimal SP for various reconstruction methods considering the same anatomy. Thus one can have a better decision on which matched sampling and reconstruction is the most effective for specific anatomy and contrast at the desired AF. Many questions regarding the best way to sample in accelerated MRI can be answered with the help of machine learning algorithms such as BASS. Learned SPs are key elements in making higher AFs available in clinical scanners for translational research.

## Conclusion

We proposed a data-driven approach for learning the sampling pattern in parallel MRI. It has a low computational cost and converges quickly, enabling the use of large datasets to optimize large sampling patterns, which is important for high-resolution Cartesian 3D-MRI and quantitative and dynamic MRI applications. The approach considers measurements for specific anatomy and assumes a specific reconstruction method. Our experiments show that the optimized SPs are different for different reconstruction methods, suggesting that matching the sampling to the reconstruction method is important. The approach improves the acceleration factor and helps with finding the best SP for reconstruction methods in various applications of parallel MRI.

## Data Availability

Matlab codes and some sample data used for training and validation are available at https://cai2r.net/resources/software/data-driven-learning-sampling-pattern. Brain dataset is available at https://fastmri.med.nyu.edu/. Complete $\text {T}_{1\rho }$-brain and $\text {T}_{1\rho }$-knee datasets are available on request.
